# Kenneth Kendler: fully human

**DOI:** 10.1192/bjb.2020.105

**Published:** 2020-12

**Authors:** Claire McKenna

**Figure:**
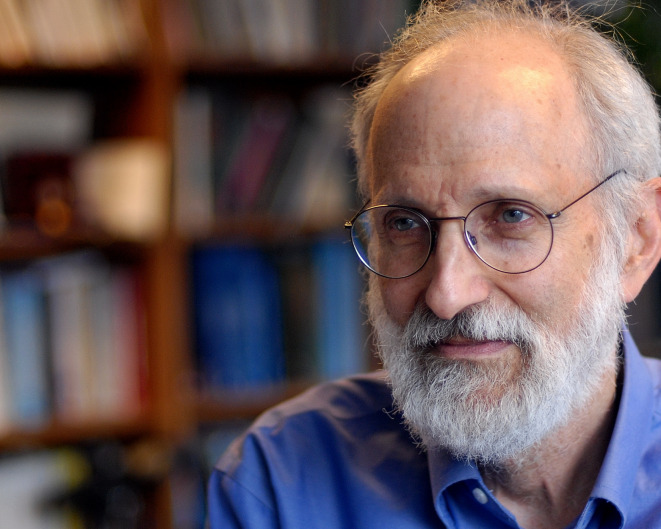

‘We shall not cease from exploration

And the end of all our exploring

Will be to arrive where we started

And know the place for the first time.’

T. S. Eliot, ‘Little Gidding’ (from *Four Quartets*), 1942

Born the son of two experimental psychologists (his father a ‘classic New York Jewish character’, his mother a ‘very intellectual, incisive’ woman who ‘overcame a lot of prejudice about being a woman in academia’), Professor Kenneth Kendler's parents were initially sceptical about his choice of a career in psychiatry: ‘In my parents’ days (they would have trained in the late 1930s and early 1940s), the smart people went on to get a PhD and the physicians were sort of second class. They basically were either psychoanalysts or they went to state hospitals and gave lots of people ECT … My father, for example, worked in the mental health system during the Second World War. So he had a lot of contact with psychiatrists and was not, on average, very impressed’. He thinks he made them proud in the end though: ‘I think eventually I convinced them one could do rigorous research in this area’.

At the time of writing, Kendler is the second most highly cited psychiatric researcher in the world. Already a renowned expert in psychiatric genetics, he diverged later in his career to become an eminent scholar in the philosophy, and latterly the history, of psychiatry. He has gone to the lengths of employing a German speaking translator for the past few years, so he can read Emil Kraepelin's writings in the original. These later interests were in part an attempt, it seems, to resolve his own *sturm und drang* about classification in psychiatry: ‘You cannot understand where we are in psychiatric nosology without understanding the historical context within which it exists’, he told me. In thinking about the arc of his career, I was reminded of the T. S. Eliot quotation in the epigraph to this piece.

His new book *Toward a Philosophical Approach to Psychiatry*^1^ is a compendium of his most important philosophical papers, as well as some of his historical and genetics papers (necessary to understand his thesis about the nature of psychiatric illness). He is an exceptionally clear and engaging writer, which makes the book an accessible read for psychiatrists who are not academic philosophers.

When I talked to Kendler via Skype in March 2020 he seemed pretty serene in his quarantine. Holed up in his home in Virginia because of COVID-19, he remained hard at work. It seems a labour of love for him. ‘That's what my kids say,’ he laughs, ‘This isn't work for you, Dad!’ His eyes wrinkle quickly and frequently into mirth throughout the interview. His formidable intelligence combines with a warm and easy manner, which transmitted through the ether. In his scholarly search for what ails us, he seems more aware than many of the humanity that gives meaning to this suffering.

Kendler's philosophical stance is poetically summarised (in a riff on Gerard Manley Hopkins) in the title of his 2015 paper, ‘The dappled nature of the causes of psychiatric illness: replacing the organic–functional/hardware–software dichotomy with empirically based pluralism’.^2^ His philosophical approach to psychiatry is very much of the pragmatic kind. He examined my more speculative questions briefly during this interview (about mysterianism for example) and, in a manner I imagined might be born of his decades long habit of daily meditation, let them float away like flotsam of the mind, finding them unhelpful to advance his project.

‘It's been a wonderful and fulfilling career,’ he says, but it is one that's far from over yet. Now aged 69, he still directs a research institute at Virginia Commonwealth University, he teaches, edits the journal *Psychological Medicine*, serves on the DSM steering committee and is very active in several psychiatric genomic consortia groups. The fifth in the series of his books (edited with Joseph Parnas and Peter Zachar), based on presentations at the philosophy of psychiatry conferences in Copenhagen that they organise, is just coming out.^3^ He has also raced out a series of papers this year based on a close reading of Kraepelin's original textbooks. ‘I can't write it fast enough,’ he says, as he chips away at the great edifice of our ignorance, one flake at a time.

This conversation has been condensed and edited.

**What are you most proud of in your prolific career?**

That's a really hard question. I think I've managed to show how careful methodological approaches can clarify how the familial influences on psychiatric illness act. I think they're quite strong, if you had to rank all the other components, but they're subtle and we tend to have very simplistic solutions, i.e. it's all genetic or it's all our environment. So, I think in the research I've done, I've laid some foundations for a kind of sound, rigorous empirical approach to these questions. And I think in the philosophy work, which has been later in my career, it's been closely related to the empirical work, of trying to provide a contextual framework for thinking clearly about these conditions and human behaviour.

Because, of course, it's our academic discipline, but we're also studying ourselves and our own behaviour. It's especially hard to think clearly and avoid previous biases. Its complexity is simply overwhelming. And although overly simplistic, one human reaction to great complexity is to develop tunnel vision. That's one way to get closure. But that's a bad way to do psychiatry. It's a bad way to do it clinically and a bad way to do it research-wise. So, I'm trying to provide a framework to think clearly and rigorously. I guess that would be the most important. I think I contributed a bit.

**I read two lovely papers^2,4^ of yours about a philosophical framework for psychiatry, which I thought were really good primers. I wanted to ask you a bit about the importance of philosophy for psychiatrists. It can sometimes seem to psychiatrists quite arcane and not necessarily applicable to their day-to-day practice.**

Well, I have to say that this is not novel. We all make philosophical assumptions about the things we do and think. And you can either examine them or you can't. But it's not that they're not there. And in psychiatry, they're legion. Like, how does the mind relate to the brain? You cannot begin to practise psychiatry without thinking about those questions. How do you interpret evidence from different layers of science? So, we might study personality theory, we might study parent–child relationships, we might study molecular genetic variants, we might study signals in the amygdala or the nucleus accumbens. How do we put those all together? How do they relate to one another?

**What you've just highlighted is that it's very difficult to do psychiatry well, because of the different styles of thinking we need. And humans tend to think in heuristics, so we tend to default to that more categorical thinking. And it's oftentimes really hard, actually, to hold in mind all of that pluralism whenever you're with patients. For example, when I'm talking about a patient to colleagues and I'm thinking about a psychosis spectrum, at the end of it the question is, well, ‘Is it psychosis or is it not?’ So, we default to a binary. I wonder whether it's the nature of our brains that we think like this?**

I think you're probably right, that goes back to my concerns that, in the face of overwhelming complexity, it's very understandable to simplify. I do believe rather deeply in pluralism, both in our research and in our clinical perspective. The best of psychiatrists, we have to wear a range of glasses: when we see someone acutely in an emergency room setting, versus in long-term therapy, those are not the same approach.

You know, ‘Beware the man with only a hammer, as he will see only a world full of nails’. And some people are like that. I grew up at a time when they were full of the hubris of biological psychiatry. And people would say things – they were not bad human beings – but they'd say things like, well, you wouldn't talk with an individual with schizophrenia, any more than you want to work with a broken computer. Just give them their medicines. And I think that's stunningly arrogant. People with schizophrenia have all the fears and wishes that we have. And providing good care is a very human process. That doesn't mean I don't try to think rigorously about dopamine pharmacology, but I think that's a stunning misdeed to only think about that. And it's not terrifically different from the psychoanalyst who used to say that thinking about the person as a working brain as well as a mind was somehow a defensive reaction. So, we're full of these in this field of mental health of ours.

**That analogy that you just used about seeing nails everywhere – similarly, I sometimes feel that as psychiatrists we're using a sledgehammer to crack a nut. Psychotropic medication is so non-specific in it's mode of action. Do you ever get that kind of cognitive dissonance?**

The short answer would be yes. In my practice, I try to develop a nuanced and collaborative approach. I mean, I didn't ever have to do very high-productivity kind of out-patient work. So, I think I was probably a bit spoiled. I hardly ever made any money at it, so I was not very good!

Yes, I agree. It's truly a challenge. And especially, you know, there are certainly quite ill individuals, with schizophrenia for example, where medical models are needed. The traditional psychological therapies really have very limited effectiveness, but what helps is to provide support, often on quite practical matters, to your patients. But again, in those situations, the level of trust is a very important thing. In caring for people with psychosis, when their psychosis starts coming, do they feel that they can call you? So, I think those human things matter a whole lot in addition to getting the psychopharmacology right.

**Did you coin the term ‘patchy reductionism’? I learned it from your writings.**

No, that's from Ken Schaffner.

**I think it's a helpful concept that we proceed in psychiatry by incrementalism.**

Correct.

**And in terms of your career progression, I've asked you about some of the high points, but do you have any wrong turns or regrets about how you've proceeded in your career at all?**

I wish I had studied way more statistics. I did a fair amount. So, for a psychiatrist, I know a good bit. As a research physician, you make a bargain. So I spent my 20s, while most of my PhD colleagues were getting trained in research, going to medical school and getting psychiatric training. It means that there are limits. I will never be as good as they are in some particular areas. I try to compensate for that with a broader vision and understanding.

I did have this naive idea when I was travelling around the back roads of Roscommon County in particular, Tyrone, Fermanagh and other places [for the Roscommon family study of schizophrenia], that we were going to crack and definitively solve the genetics of schizophrenia. And that was certainly naive. I was naive along with many other people. But we're making real progress now, real progress. But it's not at all simple.

**I was fascinated to read that you'd considered graduate school in religious studies at one point. Your positivist approach to psychiatry and then theology are quite different approaches.**

I continued with my Biblical studies throughout my adult life. I meet with a nearby Rabbi on most weekends for an hour and a half of study.

I got very interested in religious studies but I think I made the right decision. But those broader issues about human existence have been an important interest to me. And I think it's not unrelated to the philosophical issues.

**How do you square the metaphysics and theology with the empiricism?**

Intellectually, I am a hard-nosed agnostic. But emotionally, there's no question that there are theistic elements within me, and that's been true my whole life, which my parents were very puzzled about. I don't really feel I need to apologise for that.

We've been working on the Book of Genesis now for about three and a half years and I find those very meaningful comments on human experience. I mean, I love Homer and I think one can learn tremendous amounts about humans in Tolstoy and others. There is a descriptive approach to the nature of human experience in high-quality poetry that I have always found to be useful. It's a different way of knowing, absolutely. But they are mutually enriching. So, I am quite intolerant of the Richard Dawkinses of the world. It's so full of hubris to feel that so much wisdom and human struggle, which has been articulated in a religious context – to sort of wipe that away with one sweep as if that's just, you know, silliness. I have very little patience for that.

**That kind of tallies with what Noam Chomsky has written – that there are limits to what we can understand with science. I have a quote from him here: ‘It is quite possible – overwhelmingly probable, one might guess – that we will always learn more about human life and human personality from novels than from scientific psychology’.**

Well that's a question of epistemology. That is the ways of knowing. That's not very different from Karl Jaspers’ ‘of explanation and understanding’ actually. It's actually very similar to that. And does clinical psychiatry rely on both of those? Absolutely. Good psychiatry is always going to involve first-person, empathic understanding and that is our *craft*.

**I worry that for people like Richard Dawkins, scientism has become a bit of a religion. Do you think that?**

Well, with the following exception. If you were to ask me what is the best way to get to know about the mechanistic features of any part of the universe, I would say science is. Now, when you're talking about the human mind and its emotions, that's a different business. So in the sense that one can have hubris that science can explain things that it's not very good at, like the meaning of life or the origin of the universe, then yes, I would agree, that's scientific hubris, perhaps.

And it's funny the way that we sometimes slip as psychiatrists. I'm certainly not comfortable with the role that society calls for us. You know, as religion has gone down for people, now it's the psychiatrist who goes on talk shows. And boy, that bothers me a lot. And then, of course, they want us to comment on political figures and all that stuff. That is a big mistake.

**It's kind of like psychiatrists have become the priests of scientism.**

That's really a misunderstanding and it's amazingly widespread.

There's this attribution to us of some special form of human wisdom. It's amazing how intelligent people tend to have these feelings. It's just so strange the things that we get projected upon us as psychiatrists.

**Were your parents religious?**

Not at all. They were very Jewish, but not at all religious. I mean, if you've seen Woody Allen movies, yes? That's my father. But they grew up at a time when being religious they thought was superstition.

**And if we were going to be psychoanalysts about this, do you think that you might have had some unconscious urge to rebel?**

Well, I don't actually think that my interest in religion was primarily rebellion. It was something much more deep. You know I read a lot of Walt Whitman and William James's *Varieties of Religious Experience* when I was a teenager. I was reading Alan Watts, reading a lot of Gary Snyder poetry and other ‘Pacific Poets’. I was trying to make sense out of life in the way that I emotionally came toward it.

**Is it mainly poetry that you read in terms of the arts?**

I certainly would read poetry more than I would read fiction nowadays. I certainly started out with it. I mean, Gary Snyder is probably my paradigmatic poet of interest, and Kenneth Rexroth.

I always have several books of poetry on my table that I read.

**What do you get out of those?**

The best kind of poems are just like little prayers, little senses of pulling on the special, the contingent, even – if you want to use the word – ‘sacred’ out of our everyday life experience, which as we know kind of rushes by us. Poetry is kind of grabbing this potent observation and thinking through the emotional implications of often very small things in our lives.
